# A large bladder stone caused by the intravesical migration of an intrauterine contraceptive device: a case report

**DOI:** 10.1186/s13256-017-1461-6

**Published:** 2017-10-22

**Authors:** W. S. L. De Silva, K. A. S. U. A. Kodithuwakku, G. U. E. Aponsu, R. M. M. Rathnayake, E. Rajasegaram

**Affiliations:** 1 Post Graduate Institute of Medicine, University of Colombo, Colombo, Sri Lanka; 2 General Surgery Unit, District General Hospital Kalutara, Nagoda, Sri Lanka

**Keywords:** Secondary bladder stone, Intrauterine contraceptive device, Transmigration of an intrauterine contraceptive device, Case report

## Abstract

**Background:**

A wide variety of complications due to the extrauterine migration of intrauterine contraceptive devices have been reported in the literature. Here we describe the case of a large bladder stone formed around a migrated Copper T380A device that was neglected and detected 15 years after insertion.

**Case presentation:**

A 48-year-old Sri Lankan woman underwent a workup for lower urinary tract symptoms and recurrent urinary tract infections over the previous 6 months. The radiographs showed a large bladder stone with an imprint of an intrauterine contraceptive device in the center of it. The device had been inserted 15 years previously. Two years after the insertion, it was considered to be missing, but our patient did not comply with the recommended follow-up. She had been completely asymptomatic until she developed lower urinary tract symptoms. After confirming the location of the stone via ultrasonography, a vesicolithotomy was performed, revealing a stone with three limbs corresponding to the shape of the Copper T380A device. The device and the threads were fully covered with the stone material. Our patient was asymptomatic following the surgery.

**Conclusions:**

A migrated intrauterine contraceptive device can act as the nidus for the formation of a secondary bladder stone. The detailed imprint of the device inside the stone and the laminated appearance of the stone material were characteristic of a secondary bladder stone formed around an intrauterine contraceptive device. Radiography and ultrasonography are adequate for the diagnosis of intravesical migration of intrauterine contraceptive devices.

## Background

Vesicolithiasis is a rare condition in an otherwise normal bladder that can be caused by outflow obstruction, chronic or recurrent infections, and intravesical foreign bodies [1]. A rare iatrogenic cause of vesicolithiasis (bladder stones) is a migrated intrauterine contraceptive device (IUCD). IUCDs are known for uterine perforation and extrauterine migration, with perforations being reported at a rate of 1.2 to 1.6 per 1000 IUCD insertions [2]. The most common sites for IUCD migration are the omentum, rectum, sigmoid colon, peritoneum, and bladder [3]. The nature of symptoms caused by the migration depends on the destination of the device. Transvesical migration usually results in lower urinary tract symptoms, even in the absence of a secondary bladder stone.

Here we have described the asymptomatic migration of an IUCD, previously considered to be missing, resulting in the formation of a large secondary bladder stone detected 15 years after the insertion. A plain X-ray was characteristic in showing the layers of stone material laid down around the limbs of the IUCD and an ultrasound scan was useful in confirming the location of the stone. Even though computed tomography is recommended for the localization of a missing IUCD, a plain radiograph and ultrasound scan was adequate in this case. The prolonged asymptomatic period observed in this case has resulted in the formation of a large stone and delayed the seeking of medical care.

## Case presentation

**Fig. 1:**
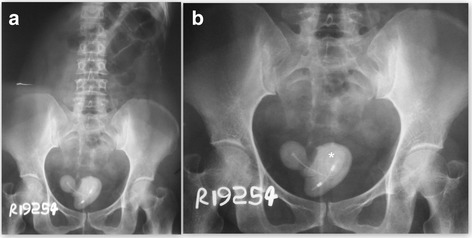
Plain radiographs. **a** The imprint of the intrauterine contraceptive device is seen in the center of the stone. **b** A magnified view of the X-ray showing the characteristic laminated appearance of the stone due to the concentric layers of stone material deposited around the intrauterine contraceptive device (*)

A 48-year-old Sri Lankan woman was referred to our general surgical clinic for the management of a bladder stone following successful treatment for a urinary tract infection complicated with upper tract involvement. She complained of intermittent nonspecific lower abdominal pain, dysuria, and hematuria over the previous 6 months. During the same period of time she had three uncomplicated urinary tract infections that were managed by her general practitioner. The urine culture grew a pure growth of *Proteus* each time. She was managed with orally administered co-amoxiclav, according to the antibacterial sensitivity report, for 1 week during each episode. She was put on nitrofurantoin as a urinary antiseptic after the third episode of urinary tract infection. On presentation for the complicated, fourth urinary tract infection, this case underwent further investigation. She was found to be septic with a heart rate of 110 beats/minute, blood pressure of 130/90 mmHg, temperature of 38.9 °C (102 °F), and respiratory rate of 20/minute. She had neutrophil leukocytosis (18 × 10^9^/ml), but her liver and renal function tests were normal. The radiographs of her kidney, ureter, and bladder showed a large bladder stone with three limbs and an imprint of a typical Copper T380A IUCD (Pregna International Ltd., Mumbai, India) in the middle of the stone (Fig. 1). An ultrasound scan of her kidney, ureter, and bladder confirmed the intravesical location of the stone and left-side pyelonephritis. Urine culture yielded a mixed growth of coliform and *Proteus*. She was managed with intravenously administered cefotaxime according to the antibacterial sensitivity report for 1 week and was continued on the nitrofurantoin until she underwent surgery.

On further inquiry, our patient indicated that she had an IUCD inserted 15 years previously, after the delivery of her third child. Two years later, the threads of the IUCD could not be found during a routine visit to a Well Woman Clinic, and it was documented as a missing IUCD. A further workup was not conducted since she did not return for a follow-up. She had forgotten about the missing IUCD and only mentioned it after being questioned. She denied having any urinary or lower abdominal symptoms until the last 6 months. She did not have any previous medical conditions. She was a housewife and had no other risk factor for urolithiasis. She had no family history of urolithiasis. Her general and abdominal examinations were otherwise unremarkable.

**Fig. 2:**
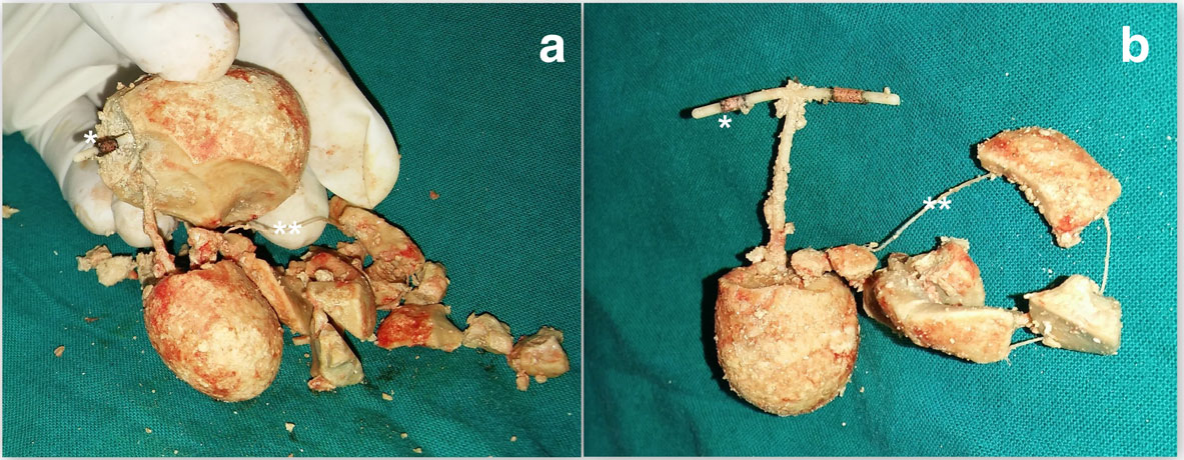
Features of the stone. **a** and **b** The copper coil (*) and the threads (**) of the intrauterine contraceptive device after breaking the stone

The diagnosis of a bladder stone formed around a migrated IUCD was made and an open vesicolithotomy was scheduled for 4 weeks later due to the large size of the stone. The vesicolithotomy was uncomplicated, and the interior of her bladder was normal. A large bladder stone with three limbs measuring 6 × 5 cm was removed, the stone was broken, and the IUCD was found inside. The three limbs of the stone were shaped to cover the three limbs of the IUCD, with the threads of the device also completely covered by the stone material (Fig. 2). Her postoperative period was uncomplicated and she was asymptomatic after the removal of the stone. At 6 months there were no further attacks of urinary tract infections.

## Discussion

The transmigration of an IUCD occurs due to traumatic primary perforation of the uterus or due to a long-term inflammatory process, the exact mechanism of which is not fully understood. The copper contained in some IUCDs can mount an inflammatory reaction that results in the contraceptive effect, but it can also be involved in the process of long-term uterine perforation and transmigration [4]. In this case, our patient could feel the threads of the IUCD during the first 2 years after insertion, but later was diagnosed as having a missing IUCD. Moreover, she did not adhere to the recommended follow-up. The perforation of the bladder wall or the mere presence of a foreign body, like an IUCD, can cause an array of lower urinary tract symptoms. Our patient did not have any symptoms over the 13 years prior to this incidence, and all of her presenting symptoms could be attributed to the presence of a large bladder stone alone. Thus, this is a case of the chronic asymptomatic migration of an IUCD into the bladder, which was discovered only after our patient became symptomatic due to the secondary stone. The imprint of the IUCD on the stone and the concentric layers of stone material noted around the IUCD in the X-ray films of our patient are characteristic of a secondary stone formed around a migrated IUCD. These two features could be seen clearly in a similar case reported by Amin and Mahmood [5]. The radiographs and ultrasonography were adequate to make the diagnosis in this case, as well as in similar cases with intravesical migration [5, 6]. However, for IUCDs lodged in other areas of the body, computed tomography may be necessary for proper localization.

The nature of the complications from a migrated IUCD depends mainly on its destination. Cases of both intraperitoneal and extraperitoneal migration locations have been reported. The omentum is the most common lodging site after intraperitoneal migration. A wide variety of complications have been reported due to such intraperitoneal IUCDs; for example, Weerasekera *et al*. reported a case of a sigmoid colocolic fistula due to an intraperitoneal IUCD [7]. Moreover, the bladder, rectum, and ureter are reported extraperitoneal IUCD migration sites. Several cases of intravesical migration have been previously reported, and a number of them have resulted in vesicolithiasis [5, 6, 8–11]. Rectal perforation [12] and ureteric erosion [13] caused by migrated IUCDs have also been reported.

In this case, the complex etiology of our patient’s bladder symptoms became clear only after performing the relevant imaging and taking a thorough history. Bladder symptoms due to an IUCD can also arise from the partial invasion of the bladder wall without transmigration [8]. Thus, a high index of suspicion should be kept in mind when managing patients with either *in situ* or missing IUCDs complaining of bladder symptoms. Moreover, this highlights the importance of arranging proper workups for all patients with missing IUCDs. The removal of a migrated IUCD after proper localization is advisable because of the unpredictability of the natural history.

## Conclusions

A migrated IUCD can act as the nidus for the formation of a secondary bladder stone. A high index of suspicion should be kept in mind when managing patients with missing IUCDs complaining of bladder symptoms. The detailed imprint of the device inside the stone and the laminated appearance of the stone material were characteristic of a secondary bladder stone formed around an IUCD. Radiography and ultrasonography are adequate for the diagnosis of intravesical migration of IUCDs.

## Abbreviation

IUCD: Intrauterine contraceptive device
